# Phytochemical Screening and Bioactivities of Cactaceae Family Members Endemic to Mexico

**DOI:** 10.3390/plants11212856

**Published:** 2022-10-26

**Authors:** Clara Angélica Rodríguez-Mendoza, Rubí Esmeralda González Campos, Ana Cecilia Lorenzo-Leal, Elizabeth Bautista Rodríguez, Genaro Alberto Paredes Juárez, Elie Girgis El Kassis, Luis Ricardo Hernández, Zaida Nelly Juárez, Horacio Bach

**Affiliations:** 1 Biotechnology Faculty, Deanship of Biological Sciences, Universidad Popular Autónoma del Estado de Puebla, 21 Sur 1103 Barrio Santiago, Puebla 72410, Mexico; 2 Division of Infectious Diseases, Department of Medicine, University of British Columbia, 2660 Oak Street, Vancouver, BC V6H 3Z6, Canada; 3 Department of degree in Medical Surgeon, Academic Secretary, Universidad de la Salud Puebla, Reforma 722, Puebla 72000, Mexico; 4 Department of Chemical Biological Sciences, Universidad de las Américas Puebla, Ex Hacienda Sta, Catarina Mártir S/N, San Andrés Cholula, Puebla 72810, Mexico; 5 Chemistry Area, Deanship of Biological Sciences, Universidad Popular Autónoma del Estado de Puebla, 21 Sur 1103 Barrio Santiago, Puebla 72410, Mexico

**Keywords:** antioxidant activity, toxicity, inflammatory response, Cactaceae, *Ferocactus*, *Mammillaria*

## Abstract

Mexico is a center of diversification for the Cactaceae family, with 69% of the species recorded as endemic. Certain members of the Cactaceae family have been chemically analyzed to relate their medicinal use with their phytochemistry. Here, the phytochemistry and bioactivity of ethanol extracts of *Ferocactus echidne*, *F. latispinus*, and *Mammillaria geminispina* were evaluated. A preliminary phytochemical analysis was performed, detecting the presence of saponins, tannins, cardiotonic glycosides, and sesquiterpene lactones. The presence of nicotinic acid in *F. echidne* and *F. latispinus* was identified by GC-MS. Other compounds found in the extracts of these three species were gentisic acid, diosmetin, chlorogenic acid, N-methyltyramide, and hordenine. The antioxidant activity was estimated with the DPPH free radical scavenging test. To determine the toxicity of the extracts, the in vivo model of *Artemia* spp. was used. In addition, the cytotoxicity of the extract was tested on C6, HaCaT, THP-1, and U937 cell lines, while the inflammatory activity was tested by measuring the secretion of cytokines using macrophage cells. The three species showed different bioactivities, including antioxidant, antimicrobial, cytotoxic, and anti-inflammatory activities. To the best of our knowledge, the results presented here are the first described for these species.

## 1. Introduction

An alternative to the integrated management of ecosystems, and their ensured maintenance, is to offer products created from native species of high economic potential and easy transformation. This goal involves the study of species other than those already exploited, such as certain kinds of cacti.

The Cactaceae family includes several species, many of which are endemic to Mexico [1]. The knowledge and use of these cacti date from the pre-Hispanic era, with records of their use as food, construction material, fences, soil fixers that prevent erosion, and fodder [2]. In addition, cacti possess a high economic value in urban environments due to their demand as ornamental plants.

Over the last 15 years, indigenous cultures and rural communities have used certain cacti species in traditional medicine. However, research on their chemical profiles and bioactivities is scarce.

Ethnobotanical studies indicate that species of the *Ferocactus* are used as antidiabetics in the Mexican folk medicine [3]. In particular, *F. latispinus* has been reported for its use as an antidiabetic and anti-inflammatory for the treatment of kidney disease [4]. On the other hand, *F. echidne* has been reported for food use [5]. Evidence of the antioxidant activity of this cactus has been reported as a reductant in the fabrication of metallic nanoparticles [6,7]. Lastly, *Mammillaria geminispina* has been reported for the treatment against epidermal excrescences associated with some types of cancers [8].

From a biochemical perspective, in addition to the Opuntioideae subfamily [9,10,11,12,13], there are species from other cactus subfamilies in which some anticancer effects have also been reported. For example, alkaloids found in *Ferocactus* ssp. were reported to reduce mammary cancer cells’ viability [14]. Moreover, the polyphenols isolated from *M. rhodantha*, *M. spinosissima*, and *M. muehlenpfordtii* showed an antiproliferative effect on mammary and cervical cancer cells [15].

In this study, the antioxidant, toxicity, anti-inflammatory, and antimicrobial activities of *F. latispinus*, *F. echidne*, and *M. geminispina* were evaluated using different models, to investigate whether these species possess any actions of medicinal importance.

## 2. Materials and Methods

### 2.1. Plant Material and Extraction

Approximately 3 kg of fresh material of each species—*F. echidne*, *F. latispinus*, and *M. geminispina*—were obtained from the Vivero Xerophila in Pachuca, Hidalgo, Mexico, in March 2020. These species were botanically classified by the biologist Lucio Caamano from the Herbarium and Botanical Garden of the Benemerita Autonomous University of Puebla, Mexico, under the registration numbers HUAP-83443 for *F. latispinus* (Haw.), HUAP-83445 for *F. echidne* (D.C.) Britton & Rose, and HUAP-83444 for *M. geminispina* (Haw).

The plant stems (without spines or roots) were cut into cubes, frozen at −80 °C for 24 h, and then lyophilized (Labconco FreeZone Console, Kansas City, MO, USA) for approximately 72 h. The resulting material was stored at −18 °C in high-density polyethylene bags until its use [15,16]. The lyophilized material was then macerated with 800 mL of ethanol (96°), with occasional agitation, at room temperature for 3 d. After filtration with cotton and a long stem funnel (Kimax), the ethanol extracts were obtained after solvent evaporation using a rotary evaporator (Büchi R-124, Flawil, Switzerland) [16].

### 2.2. Determination of Antioxidant Capacity

The free radical 1,1-diphenyl-2-picrylhydrazil (DPPH) purification test was used to determine the antioxidant activity. A stock solution of 20 mg of the extract in 2 mL of ethanol was used to prepare the final concentrations of 0.10, 0.15, 0.20, 0.25, 0.30, 0.35, and 0.40 mg/mL. An ascorbic acid solution (5 mg/mL) was used as a positive control, whereas 300 μM DPPH solution and ethanol were used as negative controls. The antioxidant activity was calculated according to the published formula [17,18]:

$$\%\;Treatment\;Activity=\frac{Absorbance\;negative\;control-Absorbance\;sample\;with\;extract}{Absorbance\;negative\;control}*100$$

The half-maximal inhibitory concentration (IC_50_, in mg/mL) was determined by the linear regression of the curve of the purification activity of each extract, as well as by the formula [16,19]:

$${\mathrm{IC}}_{50}=\frac{50-b}{a}$$

where *a* is the slope of the line and *b* is the coordinate to the origin. These estimates were made with the Microsoft Excel 2016^®^ program.

### 2.3. Phytochemical Screening

Aliquots of approximately 20 mg of each ethanol extract in 1 mL of ethanol were used to test the chemical groups detailed below.

#### 2.3.1. Alkaloids

A suspension of ethanol extract in 1% HCl was prepared. After filtration, aliquots were used to measure the levels of alkaloids using the Mayer, Wagner, and Dragendorff reagents. If a precipitate was observed, the test was considered positive for alkaloids [20].

#### 2.3.2. Test for Saponins

The ethanol extract (20 mg) was mixed with hot distilled water with vigorous stirring for 3 to 5 min. The test was considered positive when a stable foam was formed in 30 min [20].

#### 2.3.3. Triterpene Assay

1 mL of acetic anhydride was mixed with 20 mg of ethanol extract from each species. After 5 min, 1–2 drops of concentrated H_2_SO_4_ were added, and the mixture was allowed to stand for 1 min. The test was considered positive if a reddish, pink, purple, green, or blue ring was observed at the interface [20].

#### 2.3.4. Tannin Tests

The ethanol extracts were filtered and tested with two or three drops of 10% FeCl_3_ and two or three drops of gelatin reagent. If a blackish-blue precipitate was present, the test was considered positive for hydrolyzable or gallic tannins; if the precipitate was brown or green, it indicated condensed tannins or catechol derivatives [20].

#### 2.3.5. Assays for Flavonoids

Four tests were performed to determine the presence of flavonoids in the extracts: (1) The ammonia test used ammonia vapors. Briefly, a filter paper strip was impregnated with an aliquot of each extract and allowed to dry at room temperature. Then, the strips were placed in a closed container containing ammonia vapors. The test was considered positive when the filter paper showed an ochre yellow color [20]. (2) The Shinoda test was performed by adding a minimum fraction of amalgamated Mg and two drops of HCl to the aliquot of the extract. The development of a red or magenta color indicated the presence of dihydroflavonols, flavonols, or flavanone. The presence of flavonones was determined by developing the orange color [20]. (3) Another test used was the Pew test. The extracts were mixed with 0.1 g of metallic Zn and two drops of 5 N HCl. If the solution turned reddish, purple, or cherry red, the test was considered positive for dihydroflavonoid. In contrast, the presence of dihydrochalcones, flavanones, and other flavonoids was determined by the appearance of a pink or brown color [17]. (4) The NaOH test determined the presence of flavonoids when the formation of a yellowish or orange color was developed upon adding two drops of 5% sodium hydroxide [20].

#### 2.3.6. Cyanogenic Glycoside Assay

A filter paper impregnated with the Grignard reagent was placed in a water bath for 30 min and dried before exposure to the extracts. The test was considered positive when the paper turned red or pink [20].

### 2.4. Cardiotonic Glycosides and Sesquiterpene Lactones Tests

To an aliquot of each ethanol extract, two or three drops of the Baljet reagent were added, and the appearance of orange or dark red colorations was considered to confirm a positive test [20].

### 2.5. Chromatographic Analysis

Gas chromatographic analysis of the ethanol extracts extracted with chloroform was performed using a gas chromatograph (Agilent Technologies 7890B) coupled with a mass spectrometer (Agilent Technologies 5977A). The capillary column used was Agilent HP-INNOwax (30 m × 320 μm × 0.25 μm). The injection volume of the samples was 1 μL. For *F. echidne*, the GC oven temperature gradient for the columns began at 80 °C for 1 min, increasing by 5 °C/min to 220 °C for 10 min. The injection port temperature was 250 °C in the split mode for a 100:1 ratio. Helium was used as the carrier gas at a pressure of 6.0696 psi and at a flow rate of 2.0 mL/min. The mass scan range was 20–500 *m*/*z*, with a solvent cut-off of 3 min. The parameters for the *F. latispinus* and *M. geminispina* samples were the same, except for the temperature gradient, which began at 80 °C, increasing by 2.5 °C/min to 220 °C for 10 min [21].

An HPLC-MS analysis was performed using an HPLC instrument (Agilent) with a diode array detector and a mass detector, both from Agilent. The compounds were separated using a Synergi Fusion-RP 80 A (4 mm, 150 × 4.6 mm, Phenomenex). The temperature of the column was maintained at 35 °C, and a solvent system composed of solvent A: H_2_O + 0.2% acetic acid and solvent B: acetonitrile + 0.2% acetic acid was used. The flow was 1 mL/min. A gradient of solvent B was used, and according to the following method: 20–95% for 0–30 min; 95% for 30–45 min; and 20% for 45.1–55 min. The injection volume was 10 μL. Samples were prepared by dissolving 100 mg of the ethanol extracts in 1 mL ethanol. The samples were centrifuged and filtrated using nylon filters of 0.22 μm. Samples were analyzed in duplicate. The analysis of the compounds was performed in positive and negative modes.

### 2.6. Brine Shrimp Assay

Brine shrimp cysts from *Artemia* ssp. were grown in artificial seawater prepared with 30 g/L of commercial sea salt in distilled water. The cysts were incubated at 25 °C with a photoperiod of 24 h. Nauplii of 24 h age were transferred to test tubes with a pipette. The nauplii were incubated with *F. echidne* extract at 10, 30, 50, 100, 250, 500, and 1000 μg/mL; *F. latispinus* extract at 1, 5, 10, 30, 50, and 100 μg/mL; and *M. geminispina* extract at 5, 10, 30, 50, 100, 250, 500 μg/mL for 24 h and a photoperiod of 12 h. The artificial sea solution was used as a blank. The following day, the number of dead nauplii was counted. The larvae were considered dead when no movement was visualized after 10 s. The experiments were conducted in triplicate. The toxicity was estimated by calculating the mean lethal concentration (LC_50_) after an incubation period of 24 h. The LC_50_ was calculated by graphing the concentration of the compounds against the percentage of dead nauplii using the Probit model [22]. The values obtained from the experiments were compared to the standard of [22]. Concentrations >1500 μg/mL were defined as harmless; 1000–1500 μg/mL was defined as non-toxic; 500–1000 μg/mL was defined as slightly toxic; 100–500 was considered as moderately toxic; 10–100 was defined as highly toxic; and 1–10 was extremely toxic.

### 2.7. Antimicrobial Assay

A microdilution assay was used to assess the antimicrobial activities of the extracts using a 96-well plate. The antifungal activity was evaluated from spores obtained according to published protocols [16]. Eight microbial strains were used in this study. Starters were prepared by growing the strains from a single colony overnight, with shaking at 37 °C using Müeller–Hilton broth (B&D). The following day, the inoculum density was adjusted to be equivalent to 0.5 on the McFarland scale by using an optical density of 0.05 at 600 nm. Fungal strains were cultured in Sabouraud broth (B&D) at 28 °C for 24 h using the same format of 96-well plates. The bacterial strains used in this study included the Gram-positive strains *Listeria monocytogenes* (ATCC BAA-679) and Methicillin-resistant *Staphylococcus aureus* (MRSA, ATCC 700698). The Gram-negative strains included *Acinetobacter baumannii* (ATCC BAA-747), *Escherichia coli* (ATCC 25922), *Klebsiella pneumoniae* clinical isolate [22], and *Pseudomonas aeruginosa* (ATCC 14210). The fungal strains included *Candida albicans* (ATCC 10231) and *Cryptococcus neoformans* (kindly provided by Dr. Karen Bartlet, University of British Columbia, BC, Canada).

Ethanol extracts were dissolved in dimethylsulfoxide (DMSO), and 20 µg/mL concentrations were evaluated in a final volume of 150 µL/well. The minimum inhibitory concentration (MIC) for each extract was determined by incubating the organisms in 96-well microplates at 37 °C for 24 h, except for the fungi, which were cultured at 28 °C for 72 h. The endpoints were determined when no turbidity in the well was observed. The experiments were performed in triplicate [22,23].

### 2.8. Cytotoxicity Test

The in vitro models used were mouse glioma C6 (ATCC CCL-107), acute myeloid leukemia U937 (ATCC/CRL 1593.2), human epidermal skin HaCaT, and human acute monocytic leukemia THP-1 (ATCC TIB-202). The medium and supplements used in this assay were sourced from Gibco. Cells were cultured in either: DMEM medium, and supplemented with 100 IU/mL penicillin, 50 µg/mL streptomycin, and 2 mM L-glutamine; or RPMI 1640, using the same supplements. All the media used as indicated above was supplemented with 5% fetal bovine serum. THP-1 monocytic cells were differentiated with 20 ng/mL of 1-phorbol-12-myristate-13-acetate (PMA) and dispensed in a 96-well plate. Cells at densities of 1 × 10^5^ cells were dispensed in 96-well plates (100 μL), cultured at 37 °C in a humidified incubator, and supplemented with 5% CO_2_. The following day, the ethanol extracts at 12.5, 25, 50, 100, 150, and 200 µg/mL were supplemented to the cell media and incubated at 37 °C for 24 h. Cells were treated with temozolomide (TMZ) for C6, staurosporine was used for THP-1, and lipopolysaccharide (LPS) at 20 μg/mL for HaCaT cells, and used as the positive controls. The colorimetric assay assessed cell viability based on the change from 3-(4,5-dimethylthiazole-2-yl)-2,5-diphenyltetrazolium (MTT) bromide to its formazan insoluble form. After the overnight exposure, 50 µL of MTT stock solution (5 mg/mL) was added 2.5 h before the end of the incubation time. Absorption at the end of the experiment was measured with a plate reader at 570 nm (Epoch, BioTek, Winooski, VT, USA). The experiments were conducted in triplicate. The IC_50_ was estimated by graphing the concentrations of the extracts against the percentage of damaged cells [21,24].

The IC_50_ was calculated by non-linear regression of the curve using the following formula [24]:

$${\mathrm{IC}}_{50}=e^{\left(\frac{50-b}{a}\right)}$$

where *a* is the slope of the line and *b* is the coordinate to the origin.

### 2.9. Inflammatory Activity

THP-1 cells were differentiated with 20 ng/mL PMA, using the same cell density in a 96-well plate. The macrophages were incubated with 10 µg/mL of each ethanol extract. The final concentration of DMSO in the wells was always less than 1%. After 18 h of incubation with ethanol extracts, the supernatants were stored at 20 °C until later use. The measurement of the pro-inflammatory interleukin-6 (IL-6) and tumor necrosis factor-alpha (TNF-α) cytokines, as well as the anti-inflammatory interleukin-10 (IL-10), were performed with commercial ELISA kits (B&D) and according to the manufacturer’s instructions. The optical density was recorded at 450 nm using a plate reader (Epoch, BioTek, Winooski, VT, USA). The experiments were carried out in triplicate. The untreated cells and DMSO were used as negative controls, and LPS (10 µg/mL, Sigma-Aldrich, St. Louis, MO, USA) was used as a positive control [16].

### 2.10. Statistical Analysis

The test used to calculate the antioxidant was the non-parametric Wilcoxon and the Kruskal–Wallis. In contrast, the Student t-test was used for cytotoxicity and inflammatory activity. These analyses were performed using GraphPad Prism version 8.0.1 for Windows, (GraphPad Software, San Diego, CA, USA). A *p*-value < 0.05 was considered statistically significant.

## 3. Results and Discussion

### 3.1. Antioxidant Capacity

The antioxidant activity showed that *F. echidne* was the extract with the highest antioxidant activity with IC_50_ of 0.42 mg/mL, and was slightly higher than the ascorbic acid used as a positive control (0.13 mg/mL) (Table 1). The other two species of cacti showed similar antioxidant activities (~1.5 mg/mL).

**Figure 1:**
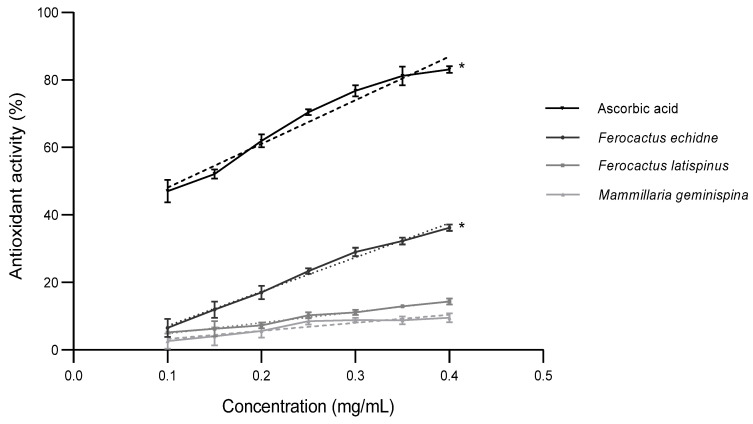
Antioxidant activity of the ethanol extracts of the three cactus species. Ascorbic acid was used as a positive control. Dashed lines represent regression analyses. Shown in the mean ± S.D. Experiments were performed in triplicate and * indicates a *p*-value < 0.05.

Other studies have reported lower IC_50_ values of the methanol extract of the callus of *Astrophytum myriostigma* with 0.19 mg/mL [26]. The most significant antioxidant activity of the DPPH radical at the concentration of 0.40 mg/mL of the ethanol extracts analyzed was presented by *F. echidne* (36.20%), followed by *F. latispinus* (14.37%), and *M. geminispina* (9.54%). Ascorbic acid, considered an excellent antioxidant, was 83.08% (Figure 1), meaning that the three species possess some antioxidant activity, although less than the ascorbic acid used as a control.

We identified the compounds gentisic acid and diosmetin in the extracts of *F. echidne*, which have been reported as antioxidant compounds [28,29], and can explain the antioxidant activities of the extract.

Other cacti have been analyzed, such as *Opuntia ficus indica* and *Opuntia robusta*. Their methanol extracts showed a higher free radical scavenging activity (66.37%) than the analyzed cacti at an extract concentration of 10 mg/mL [30]. Likewise, pulp extracts from the stems of the Brazilian cactus *Melocactus zehntneri* also showed a depurating activity of DPPH with approximately 60% of activity [31]. Specifically, for the genus *Mammillaria*, lower values ranging between 4–10 mg of Trolox equivalent/g sample with ethanol extracts of different species of that genus were reported [25].

### 3.2. Preliminary Phytochemical Analysis

Analysis of the secondary metabolites showed that the ethanol extract of *F. echidne* contains saponins, cardiotonic glycosides, and sesquiterpene lactones for *F. latispinus*; and tannins for *M. geminispine* (Table 2). These results are consistent with compounds reported by [25], who identified tannins in four species of the genus *Mammillaria*. On the other hand, an abundance of alkaloids and flavonoids was identified in the ethanol extract of *M. uncinate* [32]. In contrast, flavonoids were identified only in *M. geminispina* [25]. Of the three species, only *F. echidne* showed the presence of alkaloids, coinciding with a previous report of secondary metabolites from *Ferocactus* sp. [14].

Some types of compounds found in the three cactus species analyzed in this work correlated with the expected bioactivity. For example, benzylysquinolinic alkaloids [33] possess cytotoxic action, as do sesquiterpene lactones [34]. In the case of *F. echidne*, which showed the presence of alkaloids in the preliminary phytochemical analysis (200 μg/mL), it decreased the viability of C6 cells after 24 h of treatment by 11.41%. On the other hand, tannins are associated with antioxidant activity, and these compounds were detected in the ethanol extracts of the three species analyzed in this study [35].

### 3.3. Chromatographic Analysis

Only nicotinic acid (niacin) was identified in the *F. echidne* and *F. latispinus* extracts using GC-MS. In contrast, in the ethanol extract of *M. geminispina*, myristic acid was identified (Supplemental Information).

Niacin has been identified in the stems of the *Opuntia ficus indica* cactus [26] and in the fruits of species belonging to the genera *Hylocereus* and *Selenicereus* [36]. Furthermore, the identification of niacin can also explain the antioxidant activity of the ethanol extracts of *F. echidne* and *F. latispinus*. Several studies have shown that the antioxidant activity of the niacin [37,38,39] is based on the modulation of the glutathione content in the cell. As a result of this increase, the cell becomes more resistant to the oxidation [40]. Niacin inhibits vascular oxidative stress, redox-sensitive genes, and monocyte adhesion to human aortic endothelial cells [40]. Other studies have reported that an increase in the niacin concentration inhibits the lipid peroxidation in the cell and also increases the level of cytoplasmatic anti-oxidant enzymes such as superoxide dismutase, catalases, and glutathione peroxidase [37,41,42].

The compound identified in the *M. geminispina* extract was myristic acid. This compound has also been identified in other cacti, such as in the seed oil of *Hylocereus polyrhizus* and *Pilosocereus gounellei* [43], in *O. ficus indica* seeds [44], and in the stems of both *O. dillenii* [45] and *M. lognimamma* [46].

Myristic acid has been reported as an anti-inflammatory compound, which has been demonstrated to increase the secretion of the anti-inflammatory cytokine IL-10 upon induction with LPS using the murine macrophage cell line J774A-1 [47]. The presence of myristic acid in *M. geminispina* may explain its anti-inflammatory activity on THP-1 macrophages described below.

The compounds nicotinic acid, gentisic acid, N-methyltyramide, and hordenine were identified in the extracts of *F. echidne* and *F. latispinus*. In addition, chlorogenic acid was identified in the extracts of *F. latispinus* and *M. geminispina*, whereas diosmetin was only identified in *F. echidne* (Table 3).

### 3.4. Brine Shrimp Assay

High LC_50_ values were obtained upon the exposure of the ethanol extracts to the brine shrimp. A value of 545.56 μg/mL was recorded when the brine shrimps were exposed to the extract of *F. latispinus*, whereas values of 1500 μg/mL were measured upon the exposure to *F. echidne* and *M. geminispine*. These values indicate that the extracts of *F. echidne* and *M. geminispine* were relatively harmless compared to that of *F. latispinus* [48]. Other cactus species have recorded different toxicity levels, except for the “extremely toxic” category using the same model. For example, a moderated toxicity (LC_50_ = 282.24 ppm) was recorded when the ethanol extract of *Pachycereus schottii* was tested in a model of *Artemia salina* [49]. Similarly, the acidified ethanol extract of *Stenocereus pruinosus* was highly toxic, with an approximated LC_50_ of 80 ppm. However, acidic ethanol extracts from the same species were mild (LC_50_ = 946.78 ppm) or moderately (LC_50_ = 152.45 ppm) toxic [49]. Another study reported an LC_50_ of 547.3 ppm when the ethanol extract of the cactus *Pilosocereinus gounellei* was assessed, indicating slight toxicity [50]. Lastly, different values of toxicity were obtained when the aqueous and ethanol extracts of *Echinopsis pachanoi* showed to be harmless (LC_50_ > 10,000 ppm) and slightly toxic (LC_50_ = 543 ppm), respectively [51].

### 3.5. Antimicrobial Activity

The ethanol extracts from the three species of cacti showed antimicrobial activity as reflected by the MIC obtained, oscillating between 200 and 2000 μg/mL (Table 4).

The bacterial strains with a lower MIC (200–300 μg/mL) were clinical *S. aureus*, clinical *P. aeruginosa*, and ATCC *P. aeruginosa*. Only the *M. geminispina* extract showed a low MIC for the two fungal strains tested. This last ethanol extract showed the highest antimicrobial activity.

Other studies have reported better results against *S. aureus* with a MIC of 125 μg/mL, but used the ethyl acetate phase of the ethanol extract of the Brazilian *Tacinga inmamoena*, with no activity against *P. aeruginosa*. Moreover, the dichloromethane phase of the same extract showed a MIC = 500 μg/mL for *E. coli* [52], while in our study, the MIC values ranged between 300–400 μg/mL for the same bacterium. In summary, the antimicrobial activity, shown by the ethanol extracts of the three species endemic to Mexico against ten bacterial and two fungal strains, suggests the presence of compounds with potential antibiotic use for important infectious diseases in the region.

### 3.6. Cytotoxicity Test

**Figure 2:**
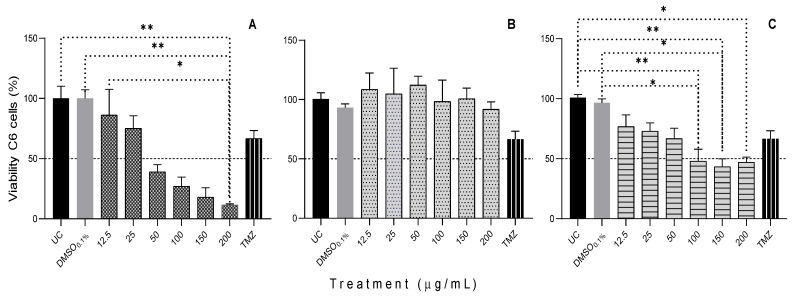
Viability of mouse glioma C6 cells in response to different concentrations of **A**: *Ferocactus echidne*; **B**: *Ferocactus latispinus*; and **C**: *Mammillaria geminispina*; after 24 h exposure. The viability test was performed using a colorimetric assay (MTT), and according to the materials and methods. UC = untreated cells (control); DMSO = Dimethylsulfoxide (control); TMZ = Temozolomide. Values are expressed as mean ± S.D. Experiments were performed in triplicate. * indicates the values with *p* < 0.05, and ** indicates the values with *p* < 0.002.

The highest concentrations of ethanolic extracts of *F. echidne* (200 μg/mL) and *M. geminispina* (100, 150, and 200 μg/mL) significantly inhibited (*p* < 0.05) the viability of the C6 mouse glioma cell line concerning the negative controls. The most significant decrease in cell viability was 24 h after the treatment of *F. echidne* was applied at its concentration of 200 μg/mL with 11.41% (*p* = 0.0012). On the contrary, the ethanolic extract of *F. latispinus* did not inhibit the viability of the C6 cell line (Figure 2).

**Figure 3:**
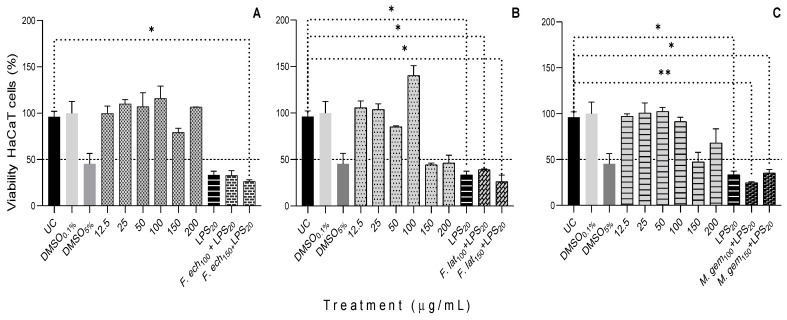
Viability of human epidermal skin HaCaT cells or keratinocytes in response to different concentrations of (**A**): *Ferocactus echidne*; (**B**): *Ferocactus latispinus*; and (**C**): *Mammillaria geminispina*; exposed for 24 h. UC = untreated cells (control); DMSO = dimethylsulfoxide (control); LPS = lipopolysaccharide (control). The viability test was performed using a colorimetric assay (MTT) and according to the materials and methods. Values are expressed as mean ± S.D. Experiments were performed in triplicate. * indicates values with *p* < 0.05, and ** indicates the values with *p* < 0.007.

For the non-cancerous cell line of human keratinocytes (HaCaT), the ethanolic extracts of *F. latispinus* and *M. geminispina* showed cytotoxicity at high concentrations (150 and 200 μg/mL) at 24 h of treatment, decreasing cell viability at less than 47%. The cells treated with LPS showed more significant toxicity (24.54%) with *M. geminispina* and 26.07% with *F. latispinus* when applying 100 and 150 μg/mL, respectively (Figure 3).

In previous research, the activity of the methanol extract of peyote *L. williamsii*, when exposed to the C6 cell line in different treatments, significantly reduced cell viability (*p* < 0.001) compared with untreated cells, depending on the time (greater than 48 h) and concentration (4.80 mg/mL) [53]. For other cell lines, significant results (*p* < 0.0001) regarding the viability of murine sarcoma cells (S180) using hydroalcoholic extracts of the columnar cactus *Cereus jamacaru* were recorded [54]. After 72 h of treatment, the extract of their fructification-stage cladodes induced a pronounced reduction in cell viability, mainly at 200, 300, 400, and 500 μg/mL. The extract of their seeds also led to a marked decrease in tumor cell viability at concentrations of 300, 400, and 500 μg/mL.

On the other hand, treatments with ethanol extracts of the cacti studied on mouse glioma C6 cells [53] produced an IC_50_ ranging from 46.50 to 712.04 μg/mL at 24 h of treatment. The lowest IC_50_ value was that of *F. echidne* (Table 5). Thus, ethanol extracts of *F. echidne* and *M. geminispina* had cytotoxic activity for C6 cells at higher concentrations. Values similar to 24 h of treatment were obtained for the ethanol extract of *Hylocereus undatus* fruits applied to chronic myeloid leukemia cells at 72 h of treatment [55].

In the case of HaCaT cells treated with different concentrations of the ethanol extracts of the three cacti, the IC_50_ values were lower than those of C6 cells. *F. latispinus* showed the highest cytotoxicity with an IC_50_ value of 6.83 μg/mL (Table 5). In the case of *Myrtillocactus geometrizans*, a species of the same subfamily as the three cacti analyzed in this study, the isolated compounds resulted in similar effects (IC_50_ of 19 to more than 200 μM) on human prostate carcinoma cells [55]. Otherwise, *O. milpa alta* polysaccharides showed a protective ability of HaCaT cells from LPS-induced (1 μg/mL) cell injury [56].

**Figure 4:**
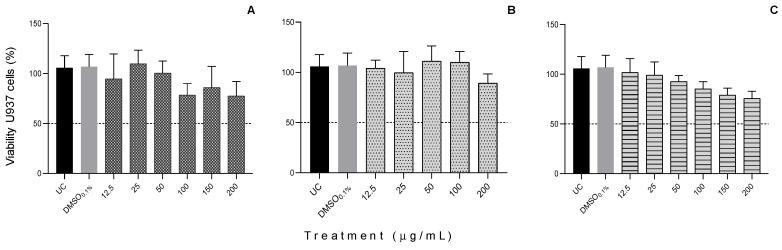
Viability of human histiocytic lymphoma U937 cells in response to different concentrations of (**A**): *Ferocactus echidne*; (**B**): *Ferocactus latispinus*; and (**C**): *Mammillaria geminispina*; after an exposure of 24 h. The viability test was performed using a colorimetric test (MTT), and according to the materials and methods. UC = untreated cells (control); DMSO = dimethylsulfoxide (control). Values are expressed as mean ± S.D. Experiments were performed in triplicate.

The samples for U937 (Figure 4) and THP-1 cells did not show cytotoxicity at concentrations lower than 200 mg/mL and 20 mg/mL, respectively. The IC_50_ values on U937 cells oscillated from 37.74 to 837.90 μg/mL at 24 h of treatment (Table 5).

Similarly, the ethanol extract of *O. stricta* showed no cytotoxic effect after 48 h on the same cell line. However, the acetone extract of this cactus at concentrations of 100 and 200 μg/mL displayed cytotoxic effects, decreasing cell viability to 46.84 and 30.16%, respectively [19]. Thus, the cancer cells’ apoptosis can be influenced by the time of application of the treatment, the dose, and the compounds of the cacti extracts, whether combined or isolated.

### 3.7. Inflammatory Activity

The inflammatory response of the ethanol extracts of *F. echidne*, *F. latispinus*, and *M. geminispine* was measured in an ex vivo model of human monocyte-derived macrophages (THP-1 cells). Results showed that the secretion of the pro-inflammatory cytokines IL-6 and TNF-α, as well as the anti-inflammatory cytokine IL-10, was not statistically significant. *F. latispinus* and *M. geminispine* had lower concentrations of the pro-inflammatory cytokines, with values of 455.10 pg/mL for IL-6, and 11.89 pg/mL for TNF-α. In contrast, *F. echidne* showed increased secretion of IL-10 (1377.03 pg/mL) compared to the other two species.

In the case of other cacti, the genus *Opuntia* has been widely studied for its anti-inflammatory response. Many species of this genus have shown a high anti-inflammatory potential through the effective suppression of IL-6 and TNF-α cytokines, inducible nitric oxide synthase (iNOS), cycle-oxygenases, and 5-lipoxygenase (5-LOX) [58]. Another study reported the anti-inflammatory activity of indicaxanthin, a fruit pigment of *O. ficus indica*, using a model of human intestinal epithelial cell line (Caco-2 cells) of chronic inflammatory bowel disease (IBD). Cells were stimulated with IL-1β, an essential cytokine initiating and amplifying the inflammatory activity of IBD. The incubation of Caco-2 cells with indicaxanthin (25 μM) and IL-1β prevented the release of pro-inflammatory cytokines IL-6 and IL-8, among other components. Based on this result, the authors suggested that administering indicaxanthin as a dietary pigment may have the potential to modulate inflammatory processes at the intestinal level [58]. Another species of cactus, *L. williamsii*, was analyzed by using its methanol extract to determine the secretion of cytokines. The results showed an increased level of the mRNA signals of IL-1, IL-6, and IL-8 in human mononuclear cells without altering their viability [53]. These cytokines are highly potent mediators for acute and chronic inflammation, and their activation may be associated with the ability of the methanol extract of this species to regulate the development of certain types of cancer [59].

## 4. Conclusions

This work has shown that three cactus species that are endemic to Mexico—*F. echidne*, *F. latispinus*, and *M. geminispina*—possess antioxidant, antimicrobial, cytotoxic, and anti-inflammatory activities. To the best of our knowledge, the bioactivities shown in this study are the first reported for these species, contributing to the knowledge and recognition of their medicinal value. Finally, it is necessary to continue analyzing these species, for the proposal of their use in different medical applications.

## Figures and Tables

**Table 1:** Antioxidant activity of extracts obtained from different species of Cactaceae expressed as mg/mL.

Species	Extract	Origen	IC_50_	Reference
*Ferocactus echidne* *F. latispinus* *Mammillaria geminispina*	Ethanol	Nursery Plant ^a^	0.42 ^c^ 1.55 ^c^ 1.57 ^c^	This work
*M. hahniana*	Ethanol	Nursery Plant ^b^	4.85 ^d^	[25]
*M. polythele*			7.16 ^d^	
*M. spinosisima*			10.60 ^d^	
*M. springlei*			6.41 ^d^	
*M. supertexta*			7.23 ^d^	
*Notocactus leninghausii*			7.16 ^d^	
*N. magnificus*			5.55 ^d^	
*N. roseoluteus*			9.52 ^d^	
*N. shlosserii*			9.59 ^d^	
*Astrophytum myriostigma*	Ethanol	Nursery Plant ^a^	3.96 ^c^	[26]
*A. myriostigma*		Callus	0.19 ^c^	
*Astrophytum capricorne*		Nursery Plant ^a^	0.30 ^c^	[27]
*Ariocarpus retusus*		Wild Plant ^a^	0.26 ^c^	
*Ariocarpus kotschoubeyanus*		Wild Plant ^a^	0.34 ^c^	
Ascorbic acid	---	---	0.13 ^c^	This work

IC_50_ = half maximal inhibitory concentration; a = Mexican; b = Egyptian; c = mg/mL sample; d = mg TE/g sample.

**Table 2:** Presence and relative abundance of secondary metabolites in the ethanol extracts of the cacti in this study.

Type of Metabolite	Reagent	Presence of the Metabolites		
		*Ferocactus echidne*	*Ferocactus latispinus*	*Mammillaria geminispina*
Alkaloids	Mayer	+	−	−
	Dragendorff	−	−	−
	Wagner	+	−	−
Saponins	Hot water	+++	+	+
Triterpenes	Acetic anhydride	−	−	−
Taninns	Iron(III) chloride	+	+	++
	Gelatin reagent	+	+	−
Flavonoids	Ammonia vapors	−	−	−
	Shinoda	−	−	−
	Pew Test	−	−	−
	NaOH	−	−	+
Cyanogenic glycosides	Grignard	−	−	−
Cardiotonic glycosides and sesquiterpene lactones	Baljet	+	+	+

Abundant (+++), moderate (++), scarce or doubtful (+), and negative (−).

**Table 3:** Identification of compounds in the ethanol extract using HPLC-MS.

Sample	[M-H]^+^	Compound	Sample	[M-H]^−^	Compound
*F. echidne*	124.0393	Nicotinic acid	*F. echidne*	150.0924	N-methyltyramide
	155.0339	Gentisic acid		164.1081	Hordenine
	301.0707	Diosmetin	*F. latispinus*	150.0924	N-methyltyramide
*F. latispinus*	124.0393	Nicotinic acid		164.1080	Hordenine
	155.0339	Gentisic acid			
	355.1024	Chlorogenic acid			
*M. geminispina*	355.1024	Chlorogenic acid			

[M-H]^+^ = masses in positive mode; [M-H]^−^ masses in negative mode.

**Table 4:** Antimicrobial activity of ethanol extracts expressed as the minimal inhibitory concentration (µg/mL).

Ethanol Extract	Bacteria										Fungi	
	Gram-Positive				Gram-Negative							
	Lm	Sa	C-Sa	MR-Sa	Ab	C-Ab	C-Kp	Ec	Pa	C-Pa	Ca	Cr
*F. echidne*	300	500	300	500	2000	300	300	300	200	300	1000	300
*F. latispinus*	500	500	300	600	2000	400	400	400	200	300	1000	1000
*M. geminispina*	500	400	300	500	2000	300	300	300	200	300	200	200

Lm = *Listeria monocytogenes*; Sa = *Staphylococcus aureus*; C-Sa = clinical *S. aureus*; MR-Sa = Methicillin-resistant *S. aureus*; Ab = *Acinetobacter baumannii*; C-Ab = clinical *A. baumannii*; C-Kp = clinical *Klebsiella pneumoniae*; Ec = *Escherichia coli*; Pa = *Pseudomonas aeruginosa*; C-Pa = clinical *P. aeruginosa*; Ca = *Candida albicans*; Cr = *Cryptococcus neoformans*.

**Table 5:** Cytotoxicity of the ethanol extracts expressed as IC_50_ in μg/mL.

Species	Extract	IC_50_	Cell Line	Reference
*Ferocactus echidne*	Ethanol stems	46.50 ^a^ 11.62 ^a^ (24 h) 83.13 ^a^	C6 HaCaT U937	This work
*Mammillaria geminispina*	Ethanol stems	121.08 ^a^ 6.77 ^a^ (24 h) 37.74 ^a^	C6 HaCaT U937	
*Hylocereus undatus*	Ethanol	>100 ^a^ (72 h)	K562	[55]
*Myrtillocactus geometrizans*	Chichipegenina	>200 ^b^ (72 h)	PC-3	[19]
	Peniocerol	19.35 ^b^ (72 h)	PC-3	
	Macdougalina	20.78 ^b^ (72 h)	PC-3	
*Opuntia stricta*	Acetone dried	46.84 ^a^ (48 h)	U937	[56]
	Ethanol fresh	118.88 ^a^ (48 h)	U937	
*Pereskia aculeata*	Methanol	>100 ^a^ (48 h)	MCF-7, HL60	[57]

a = μg/mL; b = μM.

## Data Availability

Not applicable.

## Supplementary Materials

The following supporting information can be downloaded at: https://www.mdpi.com/article/10.3390/plants11212856/s1, Figure S1. Comparison of the chromatograms of the ethanol extract of *Ferocactus echidne* and niacin. Figure S2. Comparison of mass spectra of the ethanol extract of *Ferocactus echidne* sample (up) with the W10N11 database (bottom), showing that it corresponds to niacin. Figure S3. Comparison of the chromatograms of the ethanolic extract of *Ferocactus latispinus* and niacin. Figure S4. Comparison of mass spectra of the ethanolic extract of *Ferocactus latispinus* sample (top) with the W10N11 da-tabase (bottom), showing that it corresponds to niacin. Figure S5. Comparison of the compound's mass spectra detected at 49.7 min of the *Mammillaria geminispina* sample (top) with the W10N11 database (bottom), showing that it corresponds to myristic acid.

## Sample Availability

Samples of the compounds are not available from the authors.
