# Supplementary material for: Phytochemical Screening and Bioactivities of Cactaceae Family Members Endemic to Mexico
Source: Plants (Basel). 2022 Oct 26;11(21):2856. doi: 10.3390/plants11212856 (PMC9653579; doi:10.3390/plants11212856)
Supplement: Supplementary file 1 [file plants-11-02856-s001.zip › plants-1891416-supplementary.pdf]

plants

SUPPLEMENTARY INFORMATION

Phytochemical Screening and Bioactivities of Cactaceae Family Members Endemic to Mexico

Clara Angélica Rodríguez-Mendoza[1], Rubí Esmeralda González Campos[1], Ana Cecilia Lorenzo-Leal[2], Elizabeth Bautista Rodríguez[1], Genaro Alberto Paredes Juárez[3], Elie Girgis El Kassis[1], Luis Ricardo Hernández[4], Zaida Nelly Juárez[5,*] and **Horacio Bach[2,*]**

[1] **Biotechnology Faculty, Deanship of Biological Sciences, Universidad Popular Autónoma del Estado de Puebla, 21 Sur 1103 Barrio Santiago, 72410, Puebla, Mexico**

[2] **Division of Infectious Disease, Department of Medicine, University of British Columbia, 2660 Oak Street, Vancouver, BC V6H 3Z6, Canada**

[3] Department of degree in Medical Surgeon. Academic Secretary. Universidad de la Salud Puebla. Reforma 722, Puebla, Puebla, 72000, Mexico

[4] **Department of Chemical Biological Sciences, Universidad de las Américas Puebla, Ex Hacienda Sta. Catarina Mártir S/N, San Andrés Cholula, 72810, Mexico**

[5] **Chemistry Area, Deanship of Biological Sciences, Universidad Popular Autónoma del Estado de Puebla, 21 Sur 1103 Barrio Santiago, 72410, Puebla, Puebla, Mexico**

Correspodence: zaidanelly.juarez@upaep.mx (Z.J.); hbach@mail.ubc.ca (H.B.)

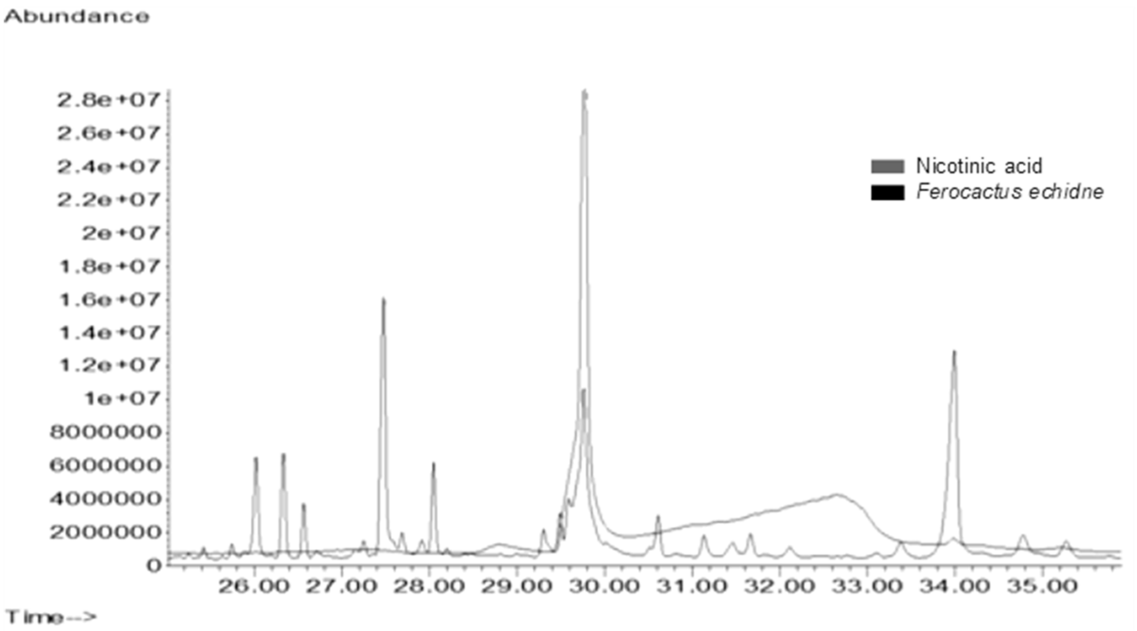

Figure S1. Comparison of the chromatograms of the ethanol extract of *Ferocactus echidne* and niacin.

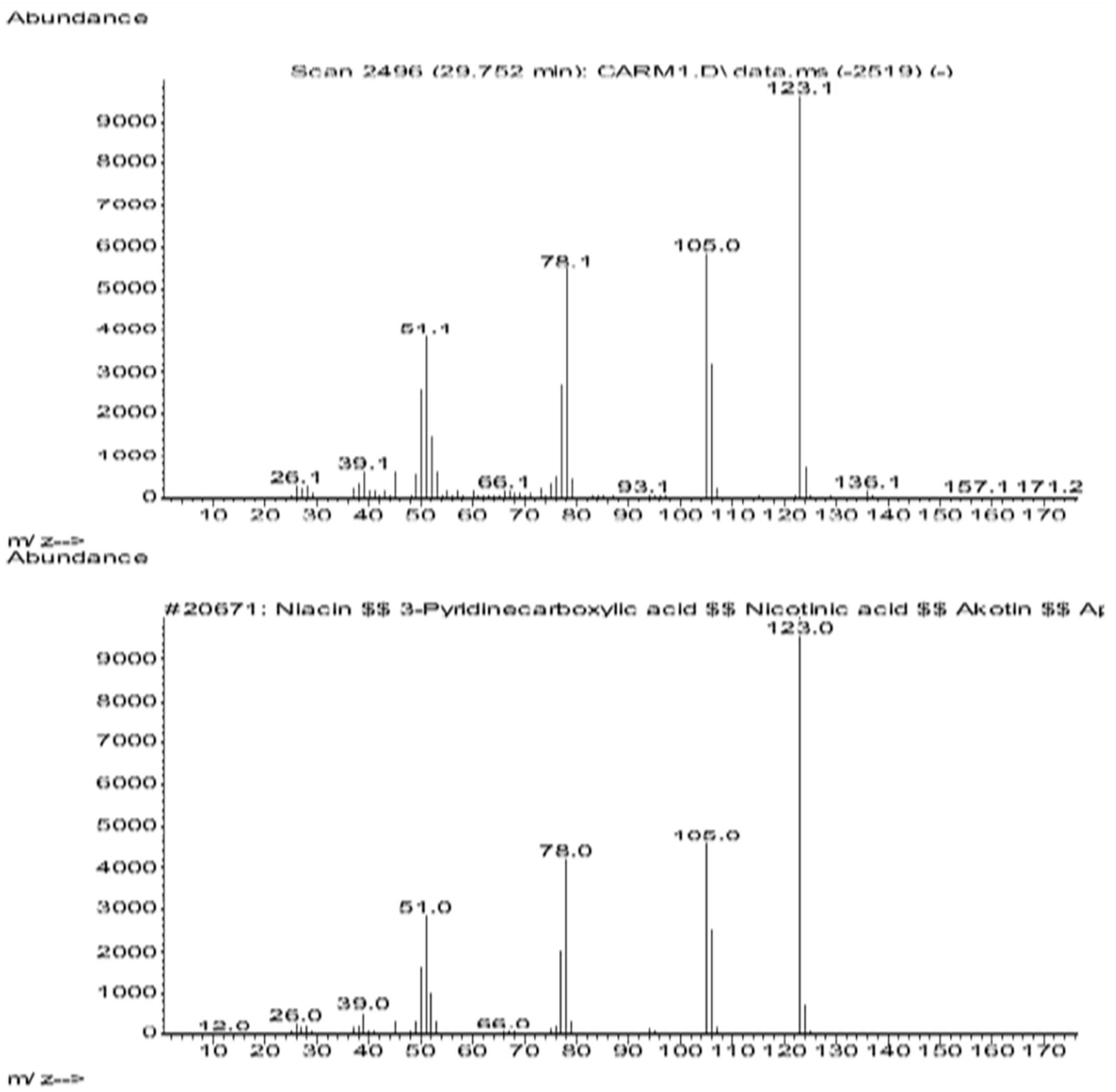

Figure S2. Comparison of mass spectra of the ethanol extract of *Ferocactus echidne* sample (up) with the W10N11 database (bottom), showing that it corresponds to niacin.

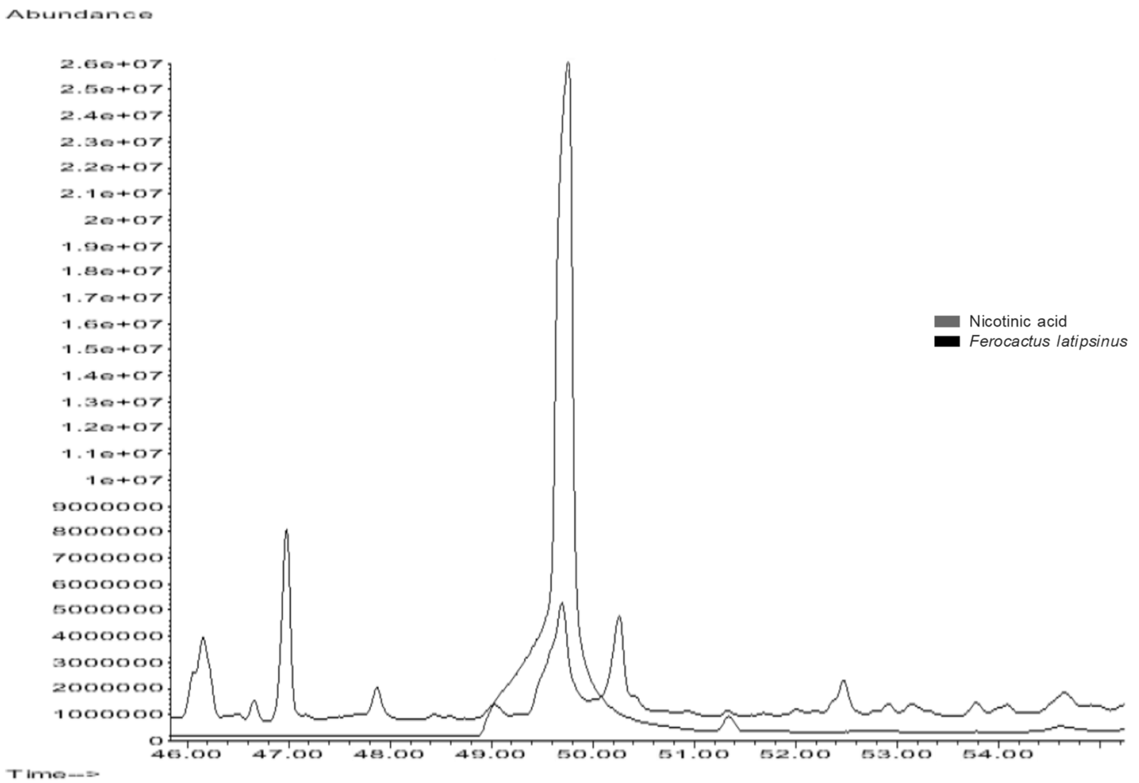

Figure S3. Comparison of the chromatograms of the ethanolic extract of *Ferocactus latispinus* and niacin.

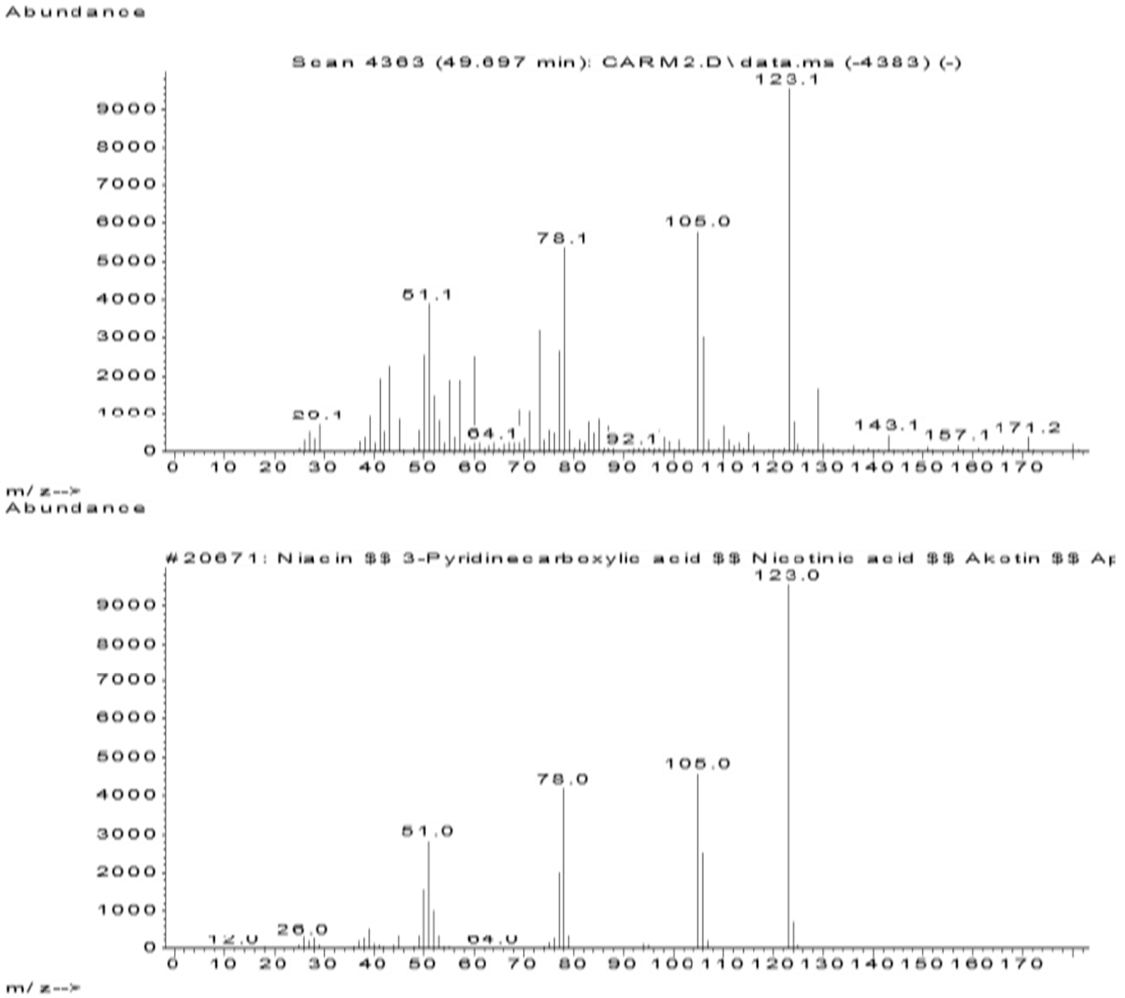

Figure S4. Comparison of mass spectra of the ethanolic extract of *Ferocactus latispinus* sample (top) with the W10N11 database (bottom), showing that it corresponds to niacin.

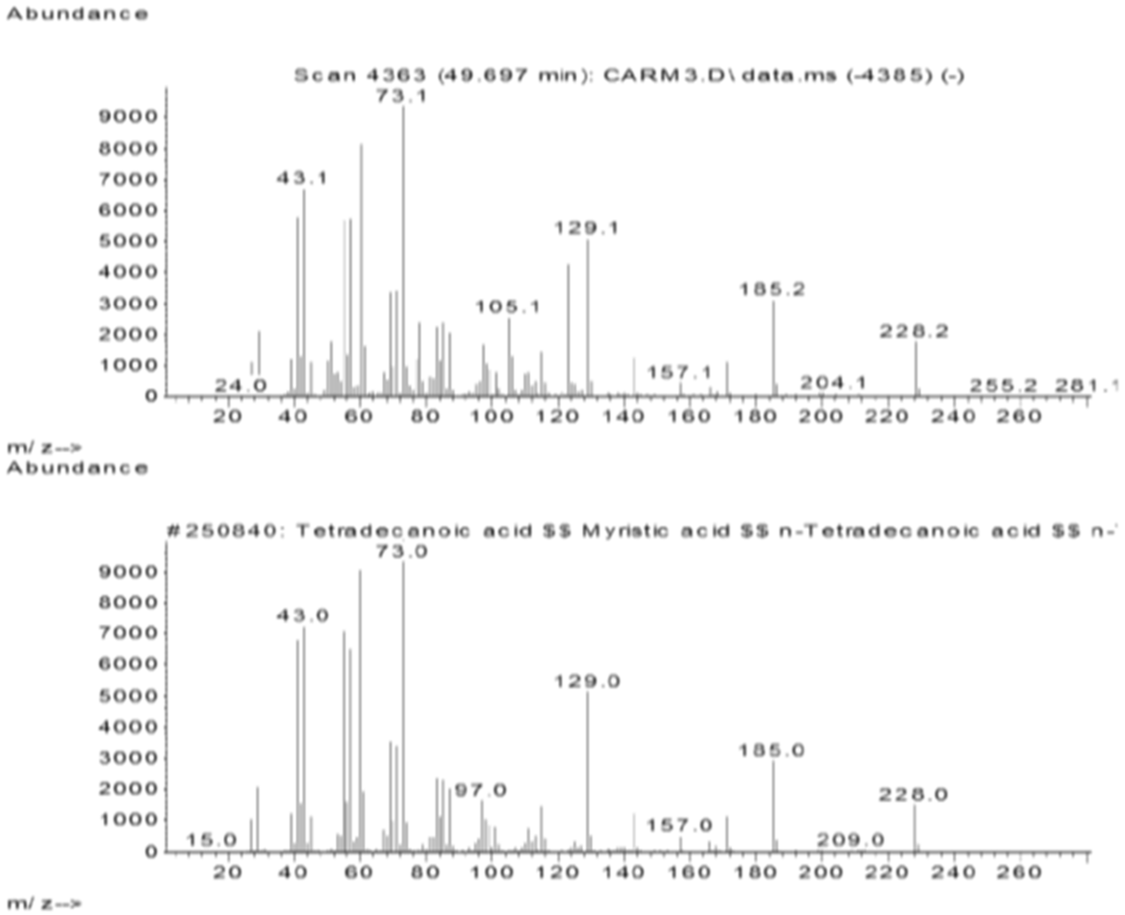

Figure S5. Comparison of the compound's mass spectra detected at 49.7 min of the *Mammillaria geminispina* sample (top) with the W10N11 database (bottom), showing that it corresponds to myristic acid.
